# Antihypertensive agents: a long way to safe drug prescribing in children

**DOI:** 10.1007/s00467-019-04314-7

**Published:** 2019-11-01

**Authors:** Nida Siddiqi, Ibrahim F. Shatat

**Affiliations:** 1 Department of Pharmacy, Sidra Medicine, Doha, Qatar; 2 Pediatric Nephrology and Hypertension, Sidra Medicine, HB. 7A. 106A, PO Box 26999, Doha, Qatar; 3 Weill Cornell College of Medicine-Qatar, Ar-Rayyan, Qatar; 4 Medical University of South Carolina, Charleston, SC USA

**Keywords:** Antihypertensive agents, Clinical trials, Drug therapy, Hypertension, Pediatric, Safety

## Abstract

Recently updated clinical guidelines have highlighted the gaps in our understanding and management of pediatric hypertension. With increased recognition and diagnosis of pediatric hypertension, the use of antihypertensive agents is also likely to increase. Drug selection to treat hypertension in the pediatric patient population remains challenging. This is primarily due to a lack of large, well-designed pediatric safety and efficacy trials, limited understanding of pharmacokinetics in children, and unknown risk of prolonged exposure to antihypertensive therapies. With newer legislation providing financial incentives for conducting clinical trials in children, along with publication of pediatric-focused guidelines, literature available for antihypertensive agents in pediatrics has increased over the last 20 years. The objective of this article is to review the literature for safety and efficacy of commonly prescribed antihypertensive agents in pediatrics. Thus far, the most data to support use in children was found for angiotensin-converting enzyme inhibitors (ACE-I), angiotensin receptor blockers (ARB), and calcium channel blockers (CCB). Several gaps were noted in the literature, particularly for beta blockers, vasodilators, and the long-term safety profile of antihypertensive agents in children. Further clinical trials are needed to guide safe and effective prescribing in the pediatric population.

## Introduction

Hypertension (HTN) in children and adolescents is defined as an average clinic measured systolic blood pressure (SBP) and/or diastolic blood pressure (DBP) ≥ 95th percentile (on the basis of age, sex, and height percentiles) [1]. Historically, pediatric HTN was considered a secondary phenomenon until proven otherwise. However, recent evidence describes primary HTN as being more likely than secondary HTN among children referred to subspecialty care for evaluation of elevated blood pressure (BP). Furthermore, the prevalence of HTN in children has been rising alongside the prevalence of obesity and increased awareness and screening among pediatricians and general practitioners. It is estimated that 3.5% of children and adolescents suffer from HTN, with prevalence as high as 25% in obese and overweight adolescents [2, 3].

In children and adolescents diagnosed with HTN, the treatment goal with non-pharmacologic and pharmacologic therapy should be a reduction in SBP and DBP to < 90th percentile or < 130/80 mm Hg, whichever is lower [1]. It is widely acceptable and recommended that lifestyle modifications should be the first-line management approach for HTN in children and adolescents. Antihypertensive medications are reserved for children with hypertensive urgencies and emergencies, evidence of target organ damage, co-existent comorbidities, and failed first-line management [1].

Clinical practice guidelines for screening and management of high BP in children and adolescents [1] recommend a stepwise therapeutic approach, starting with a single medication at the low end of the dosing range, and increasing every 2 to 4 weeks until BP is controlled (< 90th percentile), the maximal dose is reached, or adverse effects occur. If BP remains uncontrolled, a second agent can be added and dose can be titrated up as with the first agent. To balance the salt and water retention that occurs with many antihypertensive medications, a thiazide diuretic may be preferred as the second agent [1].

As is the case with many other pediatric chronic illnesses, clinicians caring for children with HTN face the dilemma of management strategies and medication utility and safety. While some concerns are related to the accuracy and reliability of BP measurements needed to confirm the diagnosis and initiate treatment, others are related to inherent physiologic, pharmacokinetic, pharmacodynamic, and daily lifestyle differences in children when compared to adults. In addition, the lack of large pediatric antihypertensive medication trials assessing dosing and safety often leads to extrapolation from adult data, raising the question of appropriate and safe prescribing. Lastly, given that antihypertensive agents are chronic medications, balancing the likelihood and risk of prolonged exposure adds to the complexity of initiating a child on these medications. For example, the use of certain classes of antihypertensives may affect bone mineral density (e.g., loop diuretics) [4], alter lipid (e.g., beta blockers and thiazide diuretics) and/or glucose metabolism (e.g., beta blockers) [5, 6], and have been correlated with an increased risk of malignancies (e.g., calcium channel blockers, angiotensin-converting enzyme inhibitors, angiotensin receptor blockers, and thiazide diuretics).

While clinicians should keep in mind the limitations to safely prescribing antihypertensive medications in children and use them only where indicated, it is also important to stress that untreated HTN has been associated with significant morbidities in children. These include but are not limited to left ventricular hypertrophy, increased markers of vascular stiffness, cognitive and learning disabilities, and faster progression of chronic kidney disease (CKD) in proteinuric hypertensive children [7–9]. However, unlike adult patients, the evidence for reduction of hard cardiovascular disease outcomes and mortality is lacking in children. Also, until now, we do not have enough evidence that treating HTN in childhood with antihypertensive medications will lead to reduction in hard clinical cardiovascular outcomes later in life; this remains to be answered in large, prospective pediatric studies.

The paradigm for pediatric antihypertensive trials has shifted in the last 20 years due to the Food and Drug Administration Modernization Act, which provided incentive for drug manufacturers to conduct clinical trials in pediatric patients. Thus, there was a surge of pediatric clinical trials by manufacturers looking to extend their patent; however, this has still left a huge gap for medications already off-patent that are still commonly prescribed in children. In this article, we aim to review the most common drug classes and pharmacologic agents used to manage HTN in pediatrics (0 to 18 years old) with an emphasis on safety (Table 1).

**Table 1:**
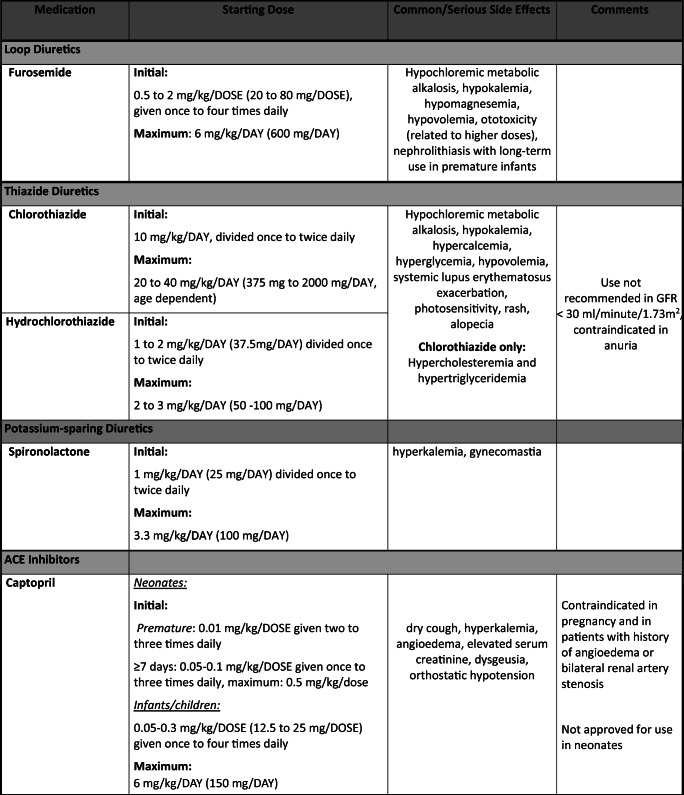

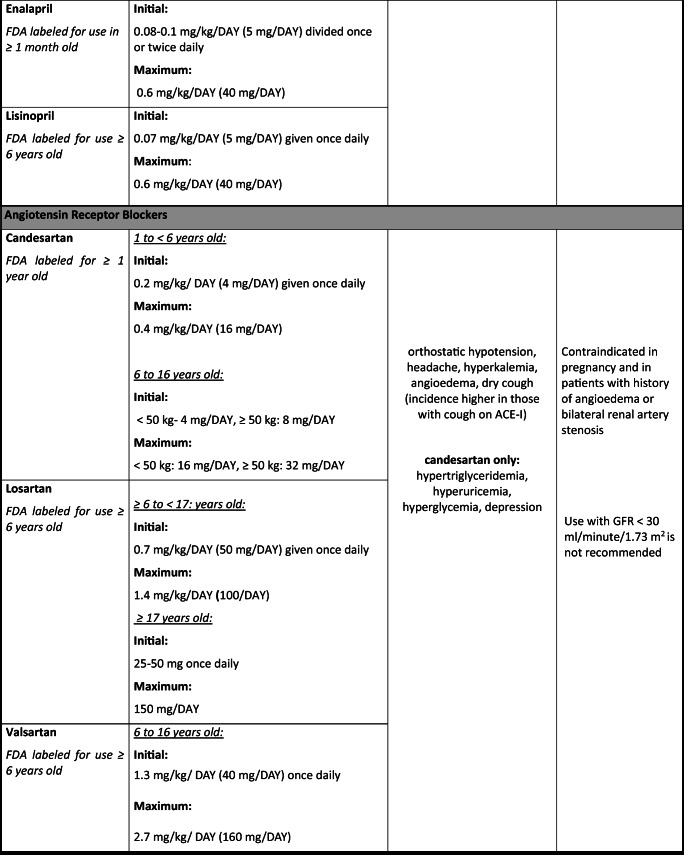

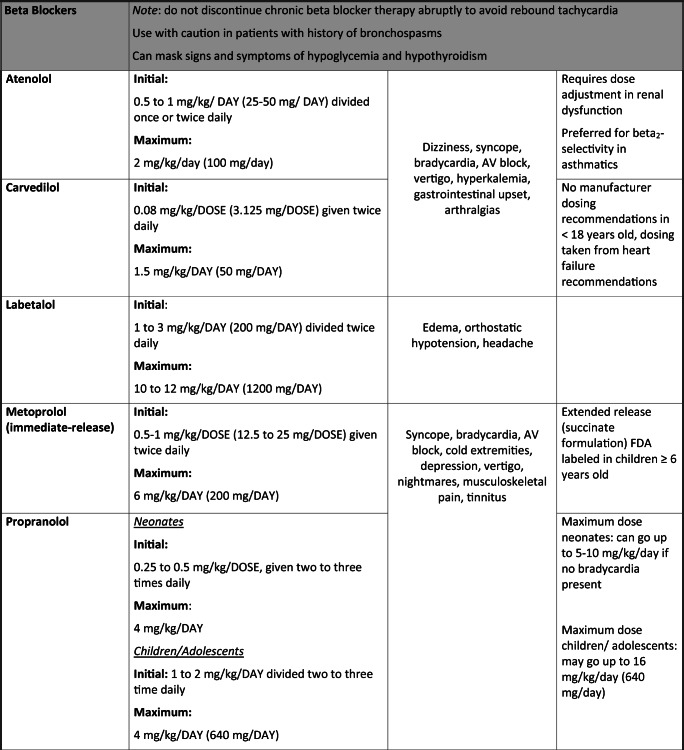

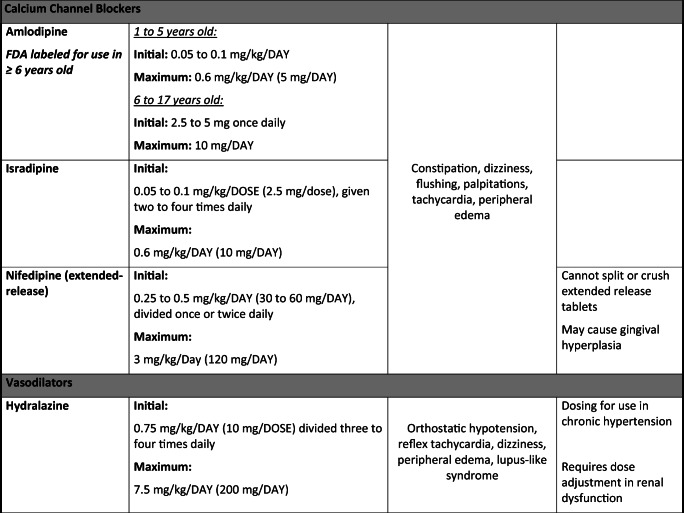
Commonly prescribed oral antihypertensive agents in pediatrics

Dosing for non-FDA-approved antihypertensive medications added for guidance and were obtained from well-established drug references, AAP guidelines [1], and published clinical trials

## Angiotensin-converting enzyme inhibitors

### Efficacy

Angiotensin-converting enzyme inhibitors (ACE-I) target the renin-angiotensin-aldosterone system (RAAS) and achieve their therapeutic effect by blocking the conversion of angiotensin I to angiotensin II, a potent vasoconstrictor [10]. Due to the inhibition of angiotensin II, ACE-I downregulate sympathetic activity, inhibit aldosterone secretion, and cause vasodilation at the efferent arteriole [10]. Furthermore, these medications inhibit the degradation of bradykinin, a vasodilator, which is also thought to contribute to the antihypertensive effects of ACE-I [11].

Being the most widely studied class, ACE-I are the most commonly prescribed antihypertensive class of medications in the pediatric patient population for both primary and secondary HTN [12]. The first study to trial use of enalapril in pediatrics included children 6 to 16 years old [13]. The authors reported a significant dose-response relationship across low, medium, and high dose groups in the initial 2-week phase (*p* < 0.001), as well as a statistically significant mean change in BP with placebo versus enalapril in the second 2-week phase (*p* < 0.003) [13]. The results of this trial led to FDA labeling of enalapril for pediatric patients. Interestingly, in the second phase the authors noted that younger patients (≤ 12 years old or ≤ Tanner stage 3) tended to have more significant rebound in BP when assigned to placebo compared to older patients [13]. Lisinopril was subsequently approved with a similar study design the following year, also demonstrating a significant dose-response relationship in children 6 to 16 years (*p* < 0.001) [14]. Other FDA-approved ACE-I in pediatrics include fosinopril and benazepril, although both approvals were also based on limited trial data in the early 2000s [15]. Captopril however, despite having the most studies published across neonates to 18 years old remains “off-label” for pediatric use.

The high prevalence of CKD as an etiology of secondary HTN in pediatrics and the reno-protective effects of ACE-I makes this drug class a desirable choice in this patient population. This likely results from the vasodilatory effects of ACE-I at the efferent arteriole, allowing for significant reduction of glomerular filtration pressure and decreased proteinuria [8]. The reno-protection is thought to be a class effect but some data postulates this may be more related to tighter BP control [16]. As reported in the ESCAPE trial, intensive BP control with ramipril aiming for targets below 50th percentile led to statistically significantly fewer patients experiencing decline in GFR by 50% or progression on end-stage kidney disease (ESKD) in intensive control group when compared to conventional control group (*p* = 0.02) [17]. In addition, patients experienced a decline in proteinuria from median 0.82 to 0.36 g protein per gram of creatinine (*p* < 0.001) [17]. It should be noted however that over 36 months proteinuria gradually returned close to baseline levels. Subsequent studies have suggested the need for ACE-I and angiotensin receptor blocker (ARB) combination therapy to prevent aldosterone “escape” and proteinuria rebound [18, 19]; however, further trials are needed to corroborate this finding.

### Safety

Despite being on the market for decades, data regarding safety of ACE-I in children remain limited. The benefits of prescribing ACE-I in the pediatric population should therefore be carefully weighed against the risks. The most commonly reported side effects with ACE-I in adults and children alike include hyperkalemia, rise in blood urea nitrogen and serum creatinine, and hypotension, with a higher incidence of hyperkalemia and elevated serum creatinine observed in neonates versus infants [20, 21]. In addition, a lower incidence of dry cough has been reported in pediatrics when compared to adult patient population [22]. Other less common adverse effects include acute kidney injury and in rare but severe cases, angioedema. Secondary to pediatric patients’ tendency towards dehydration in the setting of other common childhood illnesses, volume depletion and decreased blood flow to the kidneys only further perpetuate the risk of acute kidney injury [23]. While effective in controlling BP in renal artery stenosis [24], ACE-I are contraindicated in patients with bilateral renal artery stenosis and renal artery stenosis of a solitary kidney due to the profound risk of developing acute kidney injury and flash pulmonary edema [25–29]. The use of RAAS blockade, particularly in unilateral renal artery stenosis, however, remains controversial. This would require individualization of therapy in each clinical scenario after weighing risks versus benefits.

Given that the majority of studies were completed as “weight-banded” dose-response studies, there remains a concern regarding the most appropriate milligram per kilogram dose in pediatric patients. In addition, inherent physiologic and pharmacogenomic differences in pediatrics may further contribute to this concern. For example, higher intrinsic plasma renin activity and angiotensin II concentration in neonates increases risk of hypotension with the introduction of ACE-I and may require significant dose reductions as well as slower dose titrations [20]. Although personalizing therapy by following renin levels has been suggested [30, 31], this practice remains limited at this time. Additionally, several studies have reported attenuated response to ACE-I in adult African-American patient cohorts [32], and this finding has been corroborated in the pediatric population [33]. It should also be noted that ACE-I use is contraindicated in pregnancy due to serious risk of congenital malformations, particularly when exposure continues past the first trimester [34]. The most commonly reported fetopathies include hypocalvaria, oligohydramnios, pulmonary hypoplasia, acute kidney injury, and CKD, in addition to other neurologic and cardiovascular complications in the fetus [35]. Thus, ACE-I should be prescribed with caution in adolescent females of child-bearing potential with emphasis on the need for close follow-up in this patient population.

A recently published population-based cohort study reported an increased risk of lung cancer with ACE-I when compared to ARBs [36]. Overall, there was a 14% increased risk of lung cancer reported in adult patients on ACE-I, with the association increasing with greater than 5 years of exposure to the drug class [36]. Possible explanations may include accumulation of bradykinin and substance P in the lungs, which may have tumorigenic potential [37, 38]. It is unclear if the risk in adults is compounded secondary to other environmental exposures, and whether this risk can therefore be extrapolated to children. However, given the long-term exposure from initiation of therapy at a young age, this risk should be carefully considered. On the contrary, there is some data to suggest a protective effect of RAAS-inhibiting medications against cancer in carriers of particular ACE genotypes [39]. Thus, further studies are needed to assess the long-term safety of ACE-I prescribing in pediatrics.

## Angiotensin receptor blockers

### Efficacy

Similar to ACE-I, ARBs also target the RAAS; however, they work by directly antagonizing angiotensin II receptors. Since angiotensin II is generated by other pathways in addition to ACE, some have postulated that inhibiting the final step of this neurohormonal pathway with ARBs may provide more efficient blockade of the cardiovascular effects of angiotensin II with fewer side effects than ACE-I [40, 41]. On the contrary, with no action on ACE, ARBs do not alter bradykinin metabolism and the vasodilatory effects from elevated bradykinin levels seen with ACE-I may be lost when utilizing an ARB.

The clinical benefits of ARBs are similar to ACE-I, including effective BP reduction and decreased proteinuria [42], and they remain an acceptable initial pharmacologic option for monotherapy in managing HTN [1]. Current FDA-approved ARBs for use in pediatrics include losartan, valsartan, candesartan, and olmesartan, with the majority receiving approval for use in > 6 years old. A study conducted by Flynn and colleagues was the first to evaluate efficacy and safety of valsartan in ages 1 to 5 years old [43]. Ninety patients with SBP ≥ 95% were assigned 2:1:2 to a 2-week, double-blind, and dose-response phase (low, medium, and high dose), followed by an additional 2-week phase of valsartan versus placebo. All three dosing groups achieved a statistically significant reduction in mean seated SBP (low dose, *p* < 0.0001; medium dose, *p* = 0.0002; high dose, *p* < 0.0001) but failed to demonstrate a linear dose-response between the three groups. A statistically significant reduction was also seen when the valsartan group was compared to placebo (*p* = 0.02). It should also be noted that the majority of patients in this study had HTN secondary to renal and urinary abnormalities (80%), consistent with typical presentation of HTN in this age group. Despite this, however, assessment of proteinuria was not an endpoint in this study [43]. Wells et al. studied valsartan in children aged 6 to 16 years old with a similar study design and found dose-dependent reductions in both sitting SBP and DBP across all three dosing groups (*p* < 0.0001) [44]. Furthermore, a study comparing valsartan and enalapril showed valsartan was non-inferior to enalapril in reducing mean seated SBP (*p* < 0.0001) [45].

Trachtman and colleagues demonstrated similar efficacy of candesartan in the CINCH trial, a 4-week, double-blinded, and placebo-controlled study of 240 patients aged 6–17 years old [46]. Statistically significant reductions of SBP were seen across low (*p* = 0.0074), medium (*p* < 0.0001), and high (*p* < 0.0001) dosing groups [46]. The CINCH investigators then published results of their parallel study assessing candesartan in 93 patients aged 1 to less than 6 years old [47]. They found a decline in SBP and DBP in all three dosing groups (*p* = 0.01 and *p* = 0.03, respectively) at 4 weeks follow-up. Notably, the authors also found a statistically significant, dose-related, 57% median decline in proteinuria with candesartan at 4 weeks follow-up (*p* = 0.007). Similar findings have also been reported with losartan, the first ARB approved for use in pediatrics, with statistically significant reductions seen in both BP and proteinuria [48, 49]. In the losartan study conducted by Shahinfar et al., the mean weight across all three dosing groups was 58.7 ± 26.5 kg (range 17–152 kg) and the mean age was 12 ± 3.1 years (range 5–16 years) [48]. Additionally, like many other pediatric antihypertensive studies, the authors used a “weight-banded” study design. For example, all patients < 50 kg in the medium dose group received 25 mg of the study drug, whether their actual weight was 20 kg or 40 kg. This raises the potential challenge in extrapolating results to infants and children as well as obese patients from studies where mean weight was closer to average adult weight, and mean age was closer to adolescence, when pharmacokinetics begin to closely reflect those of adults.

Olmesartan is the most recently studied and approved ARB for use in the pediatric patient population. A study assessing the palatability of various ARBs found that more children also preferred the taste of olmesartan over irbesartan, losartan, telmisartan, and valsartan [50]. The AESOP study group conducted a large trial for use of olmesartan in 302 patients aged 6 to 16 years old [51]. In addition to a typical dose-stratified study design, this trial was unique in that the investigators divided the patients into two cohorts, with cohort A consisting patients of various races (including blacks) and cohort B consisting of black patients only. Both cohorts demonstrated a statistically significant, dose-dependent reduction in seated SBP (cohort A, *p* = 0.0008; cohort B, *p* = 0.0032) and seated DBP (cohort A, *p* = 0.0026; cohort B, *p* = 0.0125) [51]. Interestingly, in both cohorts, patients switched to placebo demonstrated an increase in seated SBP compared to patients that remained on olmesartan; however, the increase in SBP was only statistically significant in cohort A (*p* = 0.0093) and not in cohort B (no *p* value reported) [51]. Although the authors considered the sample size too small to assess statistical significance, this finding may also suggest that the diminished response to renin-angiotensin blocking agents seen in black adults may also be true in black children [52, 53].

Lastly, it would be worth noting that irbesartan failed to obtain FDA approval for pediatric HTN due to lack of efficacy.

### Safety

One potential advantage of ARBs over other antihypertensives, particularly ACE-I, may be once daily dosing across all agents within the drug class, improving compliance in an already challenging patient population. All trials have reported ARBs as safe for use in pediatrics, with the most common adverse effects found to be headache, dizziness, and diarrhea [46–48, 51]. Other adverse effects expected with ARBs are closely related to those seen with ACE-I, given that both agents work on the RAAS. Similar to ACE-I, hyperkalemia, hypotension, and rise in blood urea nitrogen and serum creatinine are all potential side effects with ARBs. However, the incidence of dry cough and angioedema is significantly lower given the limited effect on bradykinin [54]. Patients transitioned to an ARB after developing dry cough or angioedema while on ACE-I must be counseled extensively on signs and symptoms should they recur. As seen with ACE-I, ARBs are also contraindicated in patients with bilateral renal artery stenosis given the risk of developing acute kidney injury, as well as in pregnancy for fetal abnormalities.

It should also be mentioned that there have been recalls of ARBs from the market secondary to nitrosamine contamination, a potential carcinogen. Valsartan was first found to have N-nitrosodimethylamine (NDMA), then N-nitroso-N-diethylamine (NDEA), followed by losartan with N-Nitroso-N-methyl-4-aminobutyric acid (NMBA); since then, this recall has been extended to other ARBs as well, namely irbesartan [55]. Although limited to medication lots that exceeded the “acceptable” daily nitrosamine exposure standard set forth by the FDA, this has led to fear of potential carcinogen exposure in providers and patients alike. The recalls only further highlight the need for closer regulation of the drug manufacturing and supply process [55].

## Aliskerin

### Efficacy

Of the newest drugs on the market and approved for pediatrics, aliskerin has shown mixed results. Aliskerin is a direct renin inhibitor that effectively decreases both renin and subsequent angiotensin activity [56]. Despite some promise in treating HTN and proteinuria in adults [57, 58], there remains a paucity of trials completed in the pediatric population.

A case report series of aliskerin use (dose range 1.7–4 mg/kg/day) in four patients (age 5–18 years old) with proteinuria refractory to other conventional therapies showed reduction in proteinuria in all patients, with adverse effects including hyperkalemia, abdominal pain, and symptomatic hypotension [59]. In addition, two patients developed renal failure; one patient experienced reversal in serum creatinine, while the other patient progressed to dialysis despite discontinuation of aliskerin [59].

A small pharmacokinetic study (*n* = 39) in pediatric hypertensive patients 6–17 years old showed a dose-dependent increase in aliskerin plasma concentrations (range 2–6 mg/kg/day) with headache, abdominal pain, and nausea as the most common, mild adverse effects [60]. The manufacturer of aliskerin (Tekturna, Novartis) then conducted a multi-center, randomized, and double-blind 8-week study in pediatric hypertensive patients aged 6–17 years old (*n* = 267) (NCT01150357). Patients were randomized to low, medium, or high doses, which ranged from 0.1 to 0.3 mg/kg/day (6.25–25 mg), 0.75 to 1.8 mg/kg/day (37.5–150 mg), and 3 to 7.5 mg/kg/day (150–600 mg), respectively. A dose-related response was noted in phase 1 (week 0–4) with the largest change from baseline mean sitting SBP seen in high dose group of − 9.03 ± 1.01 versus − 5.54 ± 0.78 and − 5.42 ± 1.3 mmHg in the low and medium groups, respectively. Adverse effects were similar to those reported in the adult population, with diarrhea and headache being the most common.

### Safety

Despite these trials, the optimal dosing range in pediatrics for aliskerin is still unclear, with significant variability in trials and reports. It should also be noted that despite theoretical implications of using aliskerin in combination with ACE-I and ARBs to treat “renin and aldosterone escape” particularly in patients with proteinuria [61, 62], studies have reported a significant increase in the adverse effect profile, namely hyperkalemia, hypotension, and renal failure especially in patients with preexisting CKD [62–64]. The impact of use in pediatric CKD, a common finding in hypertensive pediatrics patients, is yet to be further evaluated. Lastly, aliskerin is contraindicated in patients less than 2 years of age. Given the limited evidence for treating HTN or proteinuria in children, more trials in the pediatric population to evaluate safety and efficacy are warranted.

## Diuretics

### Efficacy

There are three primary classes of diuretics that target various portions of the renal tubule. Loop diuretics are considered the most potent in action and work by inhibiting the sodium-potassium-chloride transport pump in the ascending loop of Henle [65]. This leads to a subsequent decrease in sodium and water reabsorption. Furosemide is most commonly used in neonates, children, and adolescents alike, with torsemide, bumetanide, and ethacrynic acid utilized less frequently in pediatrics. Thiazide diuretics, namely hydrochlorothiazide and chlorothiazide, also inhibit sodium reabsorption but target the distal renal tubules [66]. Although thiazide-like diuretics (chlorthalidone and metolazone) exhibit a similar mechanism of action, they are prescribed much less frequently in children. Lastly, potassium-sparing diuretics, including triamterene, amiloride, eplerenone, and primarily spironolactone, exert diuretic effect by antagonizing aldosterone in the distal tubule, causing increased sodium and water excretion into the urine while leaving behind potassium and hydrogen ions [67]. The overall resulting natriuretic effect and decrease in extracellular volume allows for reduction in BP.

Despite a lack of robust pediatric studies likely due to no financial incentives on non-patented drugs, the Clinical Practice Guideline for Screening and Management of High Blood Pressure in Children and Adolescents considers thiazide diuretics an acceptable initial pharmacologic option as monotherapy for managing HTN [1]. For long-term BP management, however, diuretics may be used in combination therapy as one would otherwise expect the RAAS and the sympathetic nervous system to compensate [68]. The only diuretic with pediatric-focused trials is eplerenone; however, the data was conflicting regarding its efficacy in treating HTN [69]. Diuretics are considered the cornerstone for treatment of monogenic forms of HTN, such as the use of triamterene and amiloride in glucocorticoid-remediable aldosteronism; the use of spironolactone, eplerenone, and amiloride in syndrome of apparent mineralocorticoid excess, triamterene and amiloride in Liddle syndrome; and the use of thiazide diuretics in Gordon syndrome [70]. In addition, diuretics may also be the preferred first-line or adjunctive agent in hypertensive patients with fluid overload or edematous conditions [71, 72].

### Safety

There are several adverse effects related to diuretic therapy that should be considered. The most common adverse effects associated with diuretics are electrolyte disturbances, primarily hyponatremia, hypo-/hyperkalemia, and hypomagnesemia. Frequent urination secondary to diuretic therapy may be difficult for children and adolescents due to interruptions in daily routine as well as sleep throughout the night, and therefore, timing of doses should be tailored to the patient’s routine. Lastly, patients particularly at higher risk for dehydration should be monitored carefully and frequently while on diuretics due to the risk of volume depletion [73].

As CKD is a common cause of pediatric HTN, it should be noted that thiazide diuretics, namely cholorthiazide and hydrochlorothiazide, are ineffective in augmenting diuresis in creatinine clearance (CrCl) < 30 mL/min/1.73 m^2^ [74]. Thiazide diuretics have also been found to contribute to systemic lupus exacerbations and parathyroid disease with prolonged use and can also lead to deranged metabolic profiles, particularly hypertriglyceridemia [75]. On the other hand, there may be a beneficial role for continuation of loop diuretics after dialysis initiation as lower rates of hospitalization, intradialytic hypotension, and lower interdialytic weight gain have been reported [76].

Loop diuretics have been identified as a primary risk factor for sensorineural hearing loss in preterm infants [77], albeit with some data showing limited statistical significance [78]. Also it should be noted that the use of loop diuretics as antihypertensive agents has been shown to correlate with poor mineral density and increased risk of fractures in adults [4, 79]. Potassium-sparing diuretics, particularly amiloride and spironolactone can increase risk of hyperkalemia, especially when used in combination with ACE-I and in the setting of renal insufficiency. Additionally, amiloride and spironolactone are notorious for the potential to cause gynecomastia in males [80], which may have a psychosocial impact in an adolescent and contribute to non-compliance; thus, eplerenone would be the preferred option in these patients. Lastly, Pederson et al. and Pottergard et al. recently reported an association between hydrochlorothiazide and risk of Merkel cell carcinoma, malignant adnexal skin tumors, and other non-melanoma skin cancers, respectively [81–84]. Although these findings have not been corroborated by other authors [83], given that hydrochlorothiazide is a known photosensitizer, routine sun protection should be emphasized in patients.

## Calcium channel blockers

### Efficacy

Among the two classes of CCBs, dihydropyridine CCBs primarily exhibit their effect as an antihypertensive by blocking the influx of extracellular calcium, which is needed for contraction of cardiac and vascular smooth muscles [85]. Inhibition of calcium flux at this step leads to peripheral arterial vasodilation, allowing for reduction in peripheral vascular resistance and BP [85]. Non-dihydropyridine CCBs (namely verapamil and diltiazem) exhibit negative chronotropic properties in addition to negative inotropy secondary to effects on the myocardium and AV node and are therefore typically reserved for patients requiring rate control [86]. For the continuity of discussing antihypertensives, this article will focus only on dihydropyridines.

The Clinical Practice Guideline for Screening and Management of High Blood Pressure in Children and Adolescents [1] includes long-acting CCBs as an acceptable first-line monotherapy agent, which primarily includes amlodipine and extended-release nifedipine. To date, amlodipine has the most literature and is the only FDA-approved CCB in pediatrics. Due to its prolonged half-life allowing for once daily dosing and a tablet form that allows for crushing and compounding of suspensions, amlodipine is the most often used CCB for maintenance therapy. In the largest pediatric study of 268 patients, a statistically significant dose-dependent SBP reduction was noted in the two dosing groups, with − 6.9 mmHg in 2.5 mg group versus placebo (*p* = 0.045) and − 8.7 mmHg in 5 mg group versus placebo (*p* = 0.005) [87]. It should be noted that despite long half-life, younger patients may require twice daily amlodipine dosing for optimal BP control [88].

Although an immediate-release formulation of nifedipine is available and effective, it is primarily recommended for acute HTN. This dosage form, however, has fallen out of favor due to larger than desired reductions in BP, development of ventricular arrhythmias, and changes in neurologic status [89, 90]. There are a limited number of studies with extended-release nifedipine and most are cross-over with other CCBs [91, 92].

CCBs may be superior in managing HTN in kidney transplant recipients when compared to other drug classes. In a systematic review and meta-analysis, CCBs were found to decrease BP, increase glomerular filtration rate, and reduce the risk for graft loss [93]. In a study of 24 post-renal transplant recipients, the authors reported similar efficacy in BP reduction with patients on extended-release nifedipine and amlodipine [91]. However, there was a 91.7% incidence of gingival hyperplasia in patients on nifedipine, with 90% reduction in symptoms with switch to amlodipine. Of note, all patients in this study were also receiving the older immunosuppressive drug cyclosporine also known to cause gingival hyperplasia, with similar trough levels reported between the two groups. Thus, it is difficult to decipher whether this side effect would be as prevalent on modern-day immunosuppressive regimens consisting of tacrolimus. In addition to limited availability of studies, the utility of extended-release nifedipine remains restricted in younger children as the extended-release tablet form cannot be crushed or split, often leaving amlodipine as the most viable option.

Felodipine’s role in treating pediatric HTN remains controversial. An earlier study of 21 patients aged 6 to 17 years old, switched from nifedipine extended-release to felodipine extended-release, showed slightly better day time reduction in DBP (*p* = 0.05), but otherwise statistically similar findings between the two groups [92]. The dose-dependent response of felodipine extended-release was evaluated by Trachtman et al. in 128 patients aged 6 to 16 years old [94]. Their study showed reduction in DBP in the felodipine extended-release 5 mg group when compared to placebo (*p* < 0.05), but no statistically significant changes in BP were noted in the 2.5 mg and 10 mg groups. Based on the results of this 2003 study, felodipine failed to obtain FDA approval labeling in pediatrics [94].

Isradipine continues to remain a viable option for acute reduction in BP [95]. Miyashita and colleagues showed a statistically significant reduction in SBP and DBP (*p* < 0.0001) with a majority of patients receiving a dose between 0.05 mg/kg and 0.1 mg/kg [96]. The highest reduction in mean arterial pressure (MAP) was noted in < 2 years old, and thus, the authors recommended a lower starting dose in this age group [96]. Given its short half-life, however, use as maintenance therapy may be limited due to frequent dosing, but stability as an oral compounded suspension serves as advantageous in pediatrics.

### Safety

The safety concerns and adverse effects of dihydropyridine CCBs appear primarily to be a class effect. The most common side effects include flushing, headache, dizziness, peripheral edema, tachycardia, nausea, vomiting, constipation, and gingival hyperplasia [86]. These are typically reversible upon discontinuation or dose reduction of the medication, making CCBs a fairly safe option in children. CCBs are metabolized by the liver via the CYP3A4 enzymatic pathway; thus, caution should be taken when prescribed concomitantly with other medications as they are highly prone to common but serious drug-drug interactions [85]. Initiation of CCBs should be done cautiously; toxicities with amlodipine have been reported at doses as low as 0.15–0.5 mg/kg, particularly in children less than 6 years old [97, 98]. Lastly, in light of conflicting data associating CCB use with cancer risk [99], it should be noted that recent adult data suggests an increased risk of pancreatic cancer in postmenopausal women [100]. The impact of these findings in the pediatric patient population however remains unclear.

One perceived advantage of CCBs may be their lack of nephrotoxicity, which makes this drug class an appealing choice as monotherapy or combination therapy in patients with kidney disease. However, it should also be noted that in adult patients with proteinuric renal diseases, studies have reported a lack of antiproteinuric effects of CCBs despite reduction in BP [101]. In addition, recent literature in pediatrics (abstract presented at the 2019 Pediatric Academic Societies Annual Meeting, Baltimore, MD) reported an increase in proteinuria in patients on dihydropyridine CCBs when compared to patients not on CCBs (*p* = 0.001) with no statistical difference in BP control (SBP, *p* = 0.420; DBP, *p* = 0.146) [102]. One proposed mechanism is that dihydropyridine CCBs block tubular protein reabsorption, leading to increased proteinuria [103]. Thus, this raises the question of the safe use of dihydropyridine CCBs as first-line therapy for HTN in children with CKD.

## Beta blockers

### Efficacy

In the most recent guidelines, beta blockers are not recommended for first-line management of HTN in pediatrics [1]. In patients unable to tolerate or still uncontrolled on ACE-I, ARBs, and/or CCBS, however, beta blockers may serve as a viable option. In addition, beta blockers may be considered for treating HTN in dialysis-dependent CKD patients and in children with migraine headaches [104, 105]. Despite being utilized for over 40 years, the beta blocker drug class has the least number of drugs FDA approved for use in pediatrics. Beta blockers work to reduce BP through both negative inotropic and chronotropic effects, thus effectively decreasing cardiac output [106]. The effect of each particular beta blocker is dependent upon specificity and selectivity to each beta-receptor type; atenolol, bisoprolol, and metoprolol are very cardioselective and block only β_1_ receptors, whereas others such as propranolol may antagonize both β_1_ and β_2_ receptors. In addition to blocking at β_1_ and β_2_ receptors, third-generation beta blockers carvedilol and nebivolol also possess vasodilatory properties, with carvedilol exhibiting α1-blocking activity [107].

Metoprolol remains the only beta blocker FDA approved for use in pediatrics. Falkner et al. reported effectiveness of metoprolol in 16 adolescents aged 12–22 years old in reducing both mean SBP (*p* < 0.001) and DBP (*p* < 0.001) with minimal side effects [108]. Patients were followed for 3 to 12 months and metoprolol dosing ranged from 100 to 200 mg daily. A more recent trial of metoprolol extended-release studied 140 patients aged 6 to 16 years old randomized to four different groups comprising: placebo, 0.2 mg/kg, 1 mg/kg, and 2 mg/kg [109]. A statistically significant decrease in BP was observed in both the 1 mg/kg (*p* = 0.027) and 2 mg/kg (*p* = 0.049) cohorts; however, this finding did not demonstrate a linear dose-response relationship [109]. It should also be noted that the majority of patients in this study were > 12 years old and obese, with limited comorbidities reported; this may not reflect typical pediatric patients with HTN where the primary cause is often of renal origin [2]. In addition, the extended-release dosage form may have limited utility in children as it can only be split in half and not crushed.

Data for propranolol, atenolol, and carvedilol in children is primarily available for varying indications such as portal hypertension, congestive heart failure, migraines, and arrhythmias, suggesting efficacy with an acceptable safety profile [110–113]. Minimal studies, however, have examined their use in HTN. Although prescribed commonly, data for propranolol in hypertensive children is limited. A small cohort of nine patients receiving propranolol at a mean dose of 2.5 mg/kg/day demonstrated a mean reduction of SBP (*p* < 0.01) and DBP (*p* < 0.01) with reports of resting bradycardia (*n* = 1) and mild, self-resolving anorexia (*n* = 1) [114]. The mean dose in this study was fairly consistent with dosing utilized today in practice [115]. Atenolol has also shown to be effective in treating essential HTN in adults [116]; however, no studies can be found in children. Labetalol, with both α- and β-blocking effects, is typically used intravenously for hypertensive emergencies, but can be used in oral form to manage chronic HTN in a patient intolerant to other beta blockers [117].

A few studies were found in the literature combining a beta blocker with a thiazide diuretic in children. A combination study of propranolol/chlorthalidone was completed in 95 children with essential HTN aged 8 to 18 years [118]. A low-dose combined drug regimen with dietary changes was used, reporting a statistically significant decrease in both SBP and DBP up to 30 months [118]. A more recent trial was done to examine the safety and efficacy of a bisoprolol-hydrochlorothiazide combination in children versus placebo [119]. Ninety-four patients aged 6–17 years were randomized to receive either combination (*n* = 62) or placebo (*n* = 32). In the treatment group, bisoprolol doses were titrated from 2.5 to 10 mg and hydrochlorothiazide dose remained at 6.25 mg. Although the authors noted a statistically significant reduction in mean sitting SBP (*p* < 0.05) and DBP (*p* < 0.05), there was no statistical difference in the percentage of patients achieving target BP control (45% treatment group versus 34% placebo, *p* = NS) [119]. Based on these results, this drug combination did not attain FDA approval for use in pediatrics.

### Safety

There are several adverse effects related to beta blocker therapy that should be carefully considered in the pediatric patient population, most often related to extent of beta-selectivity of the drug prescribed [120]. Beta blockers with β_2_ blockade activity should be cautiously prescribed in patients with history of asthma due to risk of bronchoconstriction from theoretical pulmonary cross-reactivity [120]. Other common adverse effects include bradycardia, fatigue, and secondary to their lipophilicity, central nervous system effects including vivid dreams and hallucinations. As beta blockers effectively reduce cardiac output and attenuate heart rate, they can extensively limit ability for exercise; thus, use of these agents is not recommended in athletes [121, 122].

There are several reports suggesting worsened glycemic control with beta blockers. The two postulated mechanisms include inhibition of insulin release secondary to pancreatic beta-receptor blockade and reduced peripheral blood flow, preventing glucose from reaching skeletal muscles and tissues to facilitate its disposal [5]. In addition, there is concern that beta blockers may mask symptoms of hypoglycemia, primarily tachycardia, in diabetic patients [5]. In theory, these potential adverse effects can be minimized by utilizing β_1_-selective blockers with α activity, such as carvedilol and nebivolol [123]. Compared to other antihypertensives, higher weight gain has been reported with beta blocker therapy, particularly with atenolol, metoprolol, and propranolol [124, 125]. Although the weight gain plateaus after the first few months, this should be taken into consideration when initiating patients on beta blockers [124, 125]. The impact of this observation in hypertensive children needs further investigation.

## Clonidine

### Efficacy

Clonidine stimulates the alpha_2_-adrenergic receptor, thus resulting in decreased sympathetic outflow leading to decreased peripheral vascular resistance, heart rate, and BP [126]. As recommended by the 2017 guidelines, use of clonidine to manage pediatric HTN should be reserved for patients unresponsive to two or more of the preferred agents [1]. It should be noted that the larger portion of current data in pediatrics comes from use of clonidine not only in hypertensive emergencies but also as an adjunct to sedation, analgesia, management of opioid withdrawal, and ADHD, often given at doses significantly lower than those used for HTN management [127, 128].

### Safety

Given its direct effect on the brain stem, one primary concern with use of clonidine in pediatrics is oversedation or central nervous system depression, which may contribute to behavioral changes in an already vulnerable patient population [129]. Other notable potential adverse effects with clonidine include bradycardia and rebound tachycardia when abruptly discontinuing therapy. It is prudent therefore to taper the dose when discontinuing clonidine [130]. In addition, the dosage form of clonidine being prescribed in children must be carefully considered. While the transdermal patches may potentially increase compliance, they are only indicated in children > 6 years old. Application of patches may also result in skin irritation and the potential for unexpected HTN if the patch falls off from the skin completely. Lastly, the practice of cutting transdermal patches to achieve pediatric dosing should be carefully evaluated. Zuppa and colleagues demonstrated a wider plasma concentration range, as well as a lower correlation between dose and plasma level, with cut versus whole patches [131]. The authors concluded that the rate and extent of absorption was less reliable with cut patches.

Methyldopa, also an α-receptor agonist, has been prescribed in children as well despite limited clinical trials to support is use in the pediatric patient population.

## Direct vasodilators: hydralazine and minoxidil

### Efficacy and safety

Hydralazine is a direct vasodilator that decreases BP by causing relaxation of the arteriolar smooth muscle [132] and is typically utilized in pediatric patients with uncontrolled BP unresponsive to two or more of the preferred agents [1]. There are insufficient randomized controlled trials examining the effects of chronic oral hydralazine versus placebo on SBP, DBP, morbidity, and mortality while treating essential HTN in both adults and pediatrics [133]. Use of intravenous hydralazine in children in with acute HTN has been found safe and effective; however, the change in BP has been variable. In addition, primary adverse effects reported include rebound tachycardia, edema, and excessive BP reduction [134]. In addition, although the risk is higher in adults, hydralazine has the potential of inducing or exacerbating systemic lupus erythematosus in 5–10% of patients [135].

Similar to hydralazine, minoxidil also acts by directly vasodilating arteriolar smooth muscle and is also considered a last-line option [136]. A single-dose study of minoxidil in pediatric patients aged 2 to 18 years on a beta blocker and diuretic showed statistically significant post-dose reduction in SBP and DBP (*p* < 0.05) [137]. The authors noted a significant decline in SBP within the first hour of patients receiving doses ≥ 0.2 mg/kg (*p* < 0.05), which was not seen in those receiving < 0.2 mg/kg, suggesting a dose-related effect. No major adverse effects were reported other than two patients who had rebound HTN and two patients with rise in serum creatinine [137]. Conversely, Puri et al. studied chronic minoxidil therapy in 16 renal disease patients aged 1 to 16 years old with refractory HTN. Patients were followed for 2 to 77 months and doses ranged from 0.05 to 1.88 mg/kg/day [137]. A statistically significant decrease in mean BP was noted with minoxidil therapy (*p* < 0.001). The authors reported several adverse effects, primarily hypertrichosis in 14/16 patients, fluid retention, and congestive heart failure [137]. In addition, anorexia in neonates has also been reported in the literature with use of minoxidil [138].

Finally, prazosin and doxazosin, both α-blockers, can also be used as part of the treatment regimens for specific causes of secondary HTN in pediatric patients, such as pheochromocytoma and paraganglioma [139, 140].

## Knowledge gaps and future directions

The knowledge gaps that have been noted and suggestions for future directions are summarized below:

- While HTN in children is increasingly recognized, the lack of financial incentives for pharmaceutical companies and the numbers of participants needed for antihypertensive medication trials remains a major challenge in studies of both old and new antihypertensive medications. The majority of studies examining the efficacy and safety of new antihypertensive medications in children are industry sponsored. Innovative study designs that require smaller sample sizes and partnerships between pediatric research consortiums and pharmaceutical companies are needed.
- Comparative studies of antihypertensive medication classes for the pediatric population are largely lacking. The superiority of certain antihypertensive medication classes (by ethnicity or patient population) shown in adults remains to be extensively corroborated in pediatric patients.
- Neonatal HTN remains poorly understood and there is a pressing need for studies in this patient population to identify the “optimal” safe and efficacious antihypertensive medication class. The current practices of using ACE-I and other antihypertensive medication classes in premature infants with developing organs may potentially carry life-long consequences. Epidemiological and longitudinal cohort studies of those neonates are needed.
- The majority of ACE-I and ARB trials were completed in pediatric patients ≥ 6 years old; thus, the effects of ACE-I/ARBs in younger patients remains unexamined in clinical trials. Although ACE-I are considered drugs of choice for proteinuria, the phenomenon of rebound proteinuria and aldosterone escape needs further review and understanding in pediatric patients. The safety of combining different medication classes to target the RAAS need to be further studied in pediatric patients and cannot be endorsed with the current available evidence.
- “Weight-banded” studies with high mean weights and ages make it difficult to extrapolate this data to mg/kg dosing in infants and children. Thus, more trials are needed following mg/kg study design that is more reflective of clinically appropriate dosing strategies in pediatric patients.
- The emerging data on the carcinogenic potential of some antihypertensive medication classes (ACE-I, ARBs, thiazides, and CCBs) is concerning given the potential cumulative medication dose/year exposure in pediatric patients. Epidemiological and longitudinal cohort studies of adults exposed to antihypertensive medications in their childhood are needed.
- Recent studies looking at the role of the immune system in the development of HTN, and others aiming at manipulating the gut microbiota to lower BP (NCT02037295) may bring new approaches and medication classes that have a “broader” safety profile [141].

## Conclusion

Due to a lack of large, well-designed pediatric safety and efficacy trials, limited understanding of pharmacokinetics in children, and unknown risk of life-long exposure to antihypertensive therapies, drug selection in treating pediatric HTN remains challenging. In recent years, significant progress has been made to study safety and efficacy of these agents in the pediatric population. Antihypertensive medications are generally safe to use in children, at least in the short term, but it is uncertain if their effects translate into improved long-term outcomes for children. Recent studies examining the role of the immune system and alteration of gut microbiota may allow for new approaches in managing HTN.
